# SPECT myocardial perfusion imaging as an adjunct to coronary calcium score for the detection of hemodynamically significant coronary artery stenosis

**DOI:** 10.1186/1471-2261-12-116

**Published:** 2012-12-04

**Authors:** Franz von Ziegler, Michaela Brendel, Christopher Uebleis, Susanne Helbig, Martin Greif, Janine Ruemmler, Christoph Becker, Marcus Hacker, Gerhard Steinbeck, Alexander Becker

**Affiliations:** 1 University of Munich, Department of Cardiology, Campus Grosshadern, Munich, Germany; 2 University of Munich, Department of Nuclear Medicine, Campus Grosshadern, Munich, Germany; 3 University of Munich, Department of Clinical Radiology, Campus Grosshadern, Munich, Germany

**Keywords:** Computed tomography coronary calcium scoring, Single photon emission computed tomography myocardial perfusion imaging, Invasive coronary angiography, Coronary artery disease

## Abstract

**Background:**

Coronary artery calcifications (CAC) are markers of coronary atherosclerosis, but do not correlate well with stenosis severity. This study intended to evaluate clinical situations where a combined approach of coronary calcium scoring (CS) and nuclear stress test (SPECT-MPI) is useful for the detection of relevant CAD.

**Methods:**

Patients with clinical indication for invasive coronary angiography (ICA) were included into our study during 08/2005-09/2008. At first all patients underwent CS procedure as part of the study protocol performed by either using a multidetector computed tomography (CT) scanner or a dual-source CT imager. CAC were automatically defined by dedicated software and the Agatston score was semi-automatically calculated. A stress-rest SPECT-MPI study was performed afterwards and scintigraphic images were evaluated quantitatively. Then all patients underwent ICA. Thereby significant CAD was defined as luminal stenosis ≥75% in quantitative coronary analysis (QCA) in ≥1 epicardial vessel. To compare data lacking Gaussian distribution an unpaired Wilcoxon-Test (Mann–Whitney) was used. Otherwise a Students *t*-test for unpaired samples was applied. Calculations were considered to be significant at a p-value of <0.05.

**Results:**

We consecutively included 351 symptomatic patients (mean age: 61.2±12.3 years; range: 18–94 years; male: n=240) with a mean Agatston score of 258.5±512.2 (range: 0–4214). ICA verified exclusion of significant CAD in 66/67 (98.5%) patients without CAC. CAC was detected in remaining 284 patients. In 132/284 patients (46.5%) with CS>0 significant CAD was confirmed by ICA, and excluded in 152/284 (53.5%) patients. Sensitivity for CAD detection by CS alone was calculated as 99.2%, specificity was 30.3%, and negative predictive value was 98.5%. An additional SPECT in patients with CS>0 increased specificity to 80.9% while reducing sensitivity to 87.9%. Diagnostic accuracy was 84.2%.

**Conclusions:**

In patients without CS=0 significant CAD can be excluded with a high negative predictive value by CS alone. An additional SPECT-MPI in those patients with CS>0 leads to a high diagnostic accuracy for the detection of CAD while reducing the number of patients needing invasive diagnostic procedure.

## Background

Coronary artery calcifications (CAC) are a highly specific marker of coronary atherosclerosis, the extent of which is directly related to the atherosclerotic plaque burden [1]. According to a large number of clinical studies CAC is a reliable non-invasive diagnostic tool for the prediction of subsequent cardiovascular events in populations with elevated conventional risk factors [2-13]. In a large observational report 10-year survival in asymptomatic patients with CAD was 99.4% in patients with a CAC score of 0 but was only 87.8% in those with a CAC score of greater than 1000 [14]. According to current guidelines in symptomatic patients calcium scoring (CS) might additionally serve as a useful filter prior to invasive coronary angiography or stress nuclear imaging due to its high sensitivity for flow-limiting CAD [15]. CAC studies including over 7,600 symptomatic patients demonstrate negative predictive values ranging from 96% to 100%, allowing for a high level of confidence that an individual in which CAC can be excluded has no obstructive disease [11,12,16]. However, specificity and diagnostic accuracy for prediction of flow-limiting stenosis in these patients is rather low [11-13]. Therefore, we hypothesize that combining the results of CS with the findings of a generally accepted functional imaging modality (such as single photon emission computed tomography [SPECT] or positron emission tomography [PET] myocardial perfusion imaging [MPI]) may enhance the prognostic value of both, resulting in benefits for patient screening and for the diagnosis and treatment of patients suspicious of having CAD [17-20]. Thus, the aim of our study was to evaluate different clinical scenarios in a research setting in which CS procedure alone or in combination with SPECT-MPI may enhance the diagnostic value of those non-invasive imaging modalities for the assessment of hemodynamically relevant CAD as detected by conventional invasive coronary angiography (ICA) in these particular patients.

## Methods

### Patients

An overall of 351 patients (mean age: 61.2±12.3 years; range: 18–94 years; 240 male patients; 111 female patients) were consecutively included into our study during 08/2005-09/2008. All patients had a clinical indication for ICA due to either non conclusive stress test before (i.e. stress echocardiography or stress electrocardiogram) or typical stable angina with Class II angina pectoris symptoms at minimum according to the Canadian Cardiovascular Society Classification (CCSC) system [21]. Exclusion criteria were: (1) acute coronary syndrome including ST-elevation myocardial infarction, non-ST elevation myocardial infarction, or unstable angina; (2) positive Troponin in blood testing; (3) unstable clinical conditions; (4) known coronary artery disease after stent implantation procedure or coronary artery bypass surgery; (5) age <18 years; (6) current pregnancy. The study was conducted at the University of Munich, Department of Cardiology, Campus Grosshadern, Munich, Germany and all patients gave written informed consent to participate. The study protocol was approved by the institutional review board and was in compliance with the Declaration of Helsinki. Table 1 gives a detailed overview of age and risk factors of all included patients. Patients were symptomatic with a mean CCSC of 2.4. Evaluation of cardiovascular risk profile revealed a moderate to high pre-test probability of CAD with an average number of 2.4 conventional cardiovascular risk factors.

**Table 1:** Baseline characteristics of all 351 individuals included in the study

	**All patients**	**Male patients**	**Female patients**
	351 (100.0%)	240 (68.4%)	111 (31.6%)
Mean age (years)	61.2 ± 12.3	60.0 ± 12.2	63.7 ± 12.2
Hypertension	236 (67.2%)	150 (62.5%)	86 (77.5%)
Family history	118 (33.6%)	85 (35.4%)	33 (29.7%)
Smoking	115 (32.8%)	88 (36.7%)	27 (18.9%)
Hyperlipidemia	196 (55.8%)	144 (60.0%)	52 (46.9%)
Adipositas	26 (7.4%)	13 (5.4%)	13 (11.7%)
Diabetes	38 (10.8%)	22 (9.2%)	16 (14.4%)
Average no. of risk factors	2.4 ± 0.9	2.5 ± 1.1	2.3 ± 1.0
Mean CCS Score	2.4 ± 0.9	2.4 ± 0.7	2.4 ± 0.8

### Coronary calcium scoring

CS procedure was performed by either using a multidetector CT scanner (Sensation 64; Siemens; Forchheim; Germany) or a dual-source CT imager (Definition; Siemens; Forchheim; Germany) in the thin-section mode. Standardized acquisition protocol for both scanner types included a tube voltage of 120 kV at a current of 80 mAs. Rotation time was 0.33 s. Detector collimation was 64x0.6 mm. Acquired images were reconstructed using a B35f Kernel, reconstruction increment was 1.5 mm resulting in a 50% overlap. Electrocardiographically triggered images were acquired at 80% of the R-R interval during one end-inspiratory breath-hold period. All images were electronically transferred to one dedicated workstation for CS evaluation (Leonardo; Siemens; Forchheim; Germany). Thereby, CAC were automatically defined as lesions with attenuations greater than 130 Hounsfield Units in more than four adjacent pixels. For quantification of CAC, the Agatston score was calculated as the product of the lesion's surface area and a weighting factor ranging from one to four, which was assigned according to the peak attenuation of the lesion [22]. All CS scans were evaluated by a physician unaware of the patients' clinical diagnoses.

### SPECT Myocardial Perfusion Imaging

A 1-day technetium 99 m methoxyisobutyl-isonitrile (Cardiolite; Bristol-Myers Squibb Medical Imaging; North Billerica; Massachusetts) stress-rest SPECT MPI protocol was used for all patients, as described previously [23]. Whenever possible patients were stressed ergometrically on a stationary bicycle; otherwise a pharmacological stress examination protocol was used. Thereby bodyweight- adjusted 0.56 mg dipyramidole/kg for an overall of 4 minutes was administered. No attenuation correction or gated SPECT acquisition was performed. Scintigraphic stress and rest images were evaluated quantitatively by using a dedicated software (AutoQUANT 6.0; Philips Medical Systems; Cleveland; Ohio) [24-26] that employs a 20-segment left ventricular model with a common five-point scoring system (0= “normal perfusion”; 5= “no perfusion”) [22,27]. Results were interpreted by two investigators blinded to CS and ICA results. The summed stress scores (SSS) and summed rest scores (SRS) of all segments were determined. The summed difference score (SDS) was calculated as the difference between the SSS and the SRS. A SDS≥4 was defined as stress-induced ischemia and counted as pathological SPECT-MPI.

### Conventional invasive coronary angiography

ICA was performed by the Judkins approach according to a standardized protocol using a 6-F catheter. Examinations were blinded to the results of CS as well as SPECT-MPI. Significant CAD was defined as luminal stenosis ≥75% in quantitative coronary analysis (QCA) in ≥1 epicardial vessel. For QCA the Quantcor QCA software package (Quantcor QCA; CAAS II; V 5.0; Pie Medical Imaging; Maastricht; Netherlands) was used. The decision for coronary intervention (either stent implantation procedure or coronary artery bypass surgery) was independently made by the examiner, who was blinded to the CS and SPECT-MPI results.

### Statistical analysis

All statistical analyses were performed using the SigmaStat software package (SigmaStat for Windows; V 3.10; Systat Software GmbH; Erkrath; Germany). To compare data lacking Gaussian distribution an unpaired Wilcoxon-Test (Mann–Whitney) was used. Otherwise a Students *t*-test for unpaired samples was applied. To compare the diagnostic power of CS alone or in combination with SPECT-MPI a ROC analysis was performed. All numbers are given in mean ± standard deviation. Calculations were considered to be significant at a p-value of <0.05.

## Results

### Coronary calcium scoring

CS procedure was performed without any complication in all patients with diagnostic image quality. Thus, none of the patients had to be excluded from further analysis. Mean coronary calcium Agatston score was 258.5±512.2 (range: 0–4213.7). In an overall of 67 patients (38 male; 29 female) coronary calcifications were excluded. In the remaining 284 patients (202 male; 82 female) calcified tissue was detected. Patients with CAC (mean age: 63.1±10.9 years; range: 31–94 years) were significantly older than patients without calcified tissue (53.0±14.3 years; range: 18–80 years; p<0.001). As expected, the Agatston score increased with elevated patient age. Additionally, female patients showed lower overall CS values.

### SPECT Myocardial Perfusion Imaging

All stress-rest SPECT-MPI examinations were performed without complications. 230 (65.4%) patients were stressed ergometrically on a stationary bicycle; and 121 (34.5%) patients, pharmacologically. In 303 (86.3%) patients, 0.8 mg of nitroglycerine was administered sublingually before injection of the radiopharmaceutical for the rest image.

A stress induced myocardial ischemia was detected in 148 patients (42.2%; 117 male; 31 female; mean age: 65.0±10.6 years; range: 35–94 years). In remaining 203 patients (57.8%; 123 male; 80 female) being significantly younger (mean age: 58.4±12.7 years; range: 18–85 years; p<0.001) stress induced ischemia was excluded.

### Coronary artery disease detection

All invasive examinations were performed without any complication. An overall of 133 patients (37.9%; mean age 65.3±10.1 years; range: 37–94 years) had significant CAD in ICA. This population consisted of 108 male (mean age: 64.2±9.2 years; range: 43–94 years) and 25 female patients (mean age: 69.9±12.4 years; range: 37–86 years). A coronary 1-vessel disease was found in 64 cases (48.1%), 35 patients (26.3%) had a 2-vessel disease, and all 3 coronary vessels were significantly diseased in 34 patients (25.6%). Stent implantation procedure was performed in 94 patients (70.7%) and remaining 39 patients (29.3%) underwent bypass surgery. Significant CAD was excluded by ICA in remaining 218 patients (62.1%; mean age: 58.7±12.8 years; range: 18–87 years) being significantly younger than patients with hemodynamically relevant CAD (p<0.001).

In 66/67 (98.5%) patients without coronary calcifications significant CAD leading to intervention was excluded by ICA. Only one 65-year old female patient had coronary 1-vessel disease leading to intervention in left anterior coronary artery stenosis (drug-eluting stent implantation procedure). 132/284 patients (46.5%) with positive CS also showed significant disease in ICA. Remaining 152 patients with detectable calcified tissue had no significant CAD. Thus, we calculated a sensitivity of 99.2%, a specificity of 30.3%, a negative predictive value (NPV) of 98.5%, and a positive predictive value (PPV) of 46.5% for calcium scoring alone with respect to significant CAD detection. Diagnostic accuracy (DA) was 56.4%. Table 2 gives a detailed overview over CS results using different thresholds.

**Table 2:** Diagnostic performance of CS using different thresholds compared to SPECT-MPI

	**Sensitivity**	**Specificity**	**PPV**	**NPV**	**DA**
CS =0	99.2%	30.3%	46.5%	98.5%	56.4%
CS <10	93.2%	52.8%	54.6%	92.7%	68.1%
CS <100	81.2%	83.5%	75.0%	87.9%	82.6%
CS <400	40.6%	95.4%	84.4%	72.5%	74.6%
SPECT-MPI	88.0%	85.8%	79.1%	92.1%	86.6%

In an overall of 117/148 patients (79.1%) with myocardial ischemia in SPECT-MPI (mean age: 65.0±10.6 years; range: 35–95 years) significant CAD was confirmed by ICA. False-positive results of SPECT-MPI were found in remaining 31/148 patients (21.0%). In an overall of 187/203 patients (92.2%) SPECT correctly excluded significant CAD. False-negative results were found in remaining 16/203 patients (7.9%). Thus, using SPECT imaging as stand alone modality for significant CAD detection revealed a sensitivity of 88%, a specificity of 85.8%, a NPV of 92.1%, and a PPV of 79.1%. DA for SPECT-MPI was 86.6%. ROC analysis of SPECT-MPI alone vs. SPECT-MPI in combination with CS revealed no significant difference; 0.869 (95%-CI 0.829-0.902) vs. 0.870 (95%-CI 0.830-0.903) (p=0.902). Mean CS in patients with ischemic myocardium in SPECT was significantly higher than in patients without inducible ischemia (518.6±676.4 vs. 69.4±192.4; p<0.05).

In patients with calcified plaque tissue SPECT-MPI detected stress induced ischemia in 145/284 patients (51.1%). Of these patients, 116 (80.0%) suffered from significant CAD as confirmed by ICA. In remaining 29 patients ICA excluded significant disease. In patients without inducible ischemia in SPECT-MPI but with positive CS (n=139) ICA confirmed exclusion of significant CAD in 123/139 (88.5%). False negative results were found in 16 patients (11.5%). Latter patients were significantly older compared to the general patients without detectable ischemia in our study population (mean age: 67.3±9.3 years vs. 58.4±12.7 years; p=0.0033), and additionally showed a significantly higher overall amount of calcified tissue (mean CS: 303.1±320.6 vs. 69.4±192.4; p<0.0001).

If coronary calcium scoring would have been used as a kind of filter before SPECT imaging, disease prevalence in this subgroup of patients with positive calcium scores would increase to 46.5% (132/284). Utilizing this approach a sensitivity of 87.9% with a specificity of 80.9% would have been calculated, and thus, NPV would be 88.5%, and PPV 80.0%. Theoretically, in 67 patients further scanning procedure would have been avoided, with one false negative result in aforementioned female patient. Adding additional 139 patients with positive CS, but negative SPECT-MPI studies an overall of 206/351 (58.7%) patients would have been prevented from invasive procedure with an overall of 17 (5.8%) false-negative results.

## Discussion

According to currently published reports only roughly one third of all heart catheterizations performed are leading to intervention [28]. To improve diagnostic yield the current guidelines of international cardiology societies suggest non-invasive tests for the detection of CAD in symptomatic patients, such as stress echocardiography with reported pooled sensitivity of 80-85%, and specificity of 85-86%, before invasive procedure [29]. Calcium scoring is a non-invasive modality which detects coronary calcifications being a surrogate marker for cardiac atherosclerosis. According to current literature the amount of calcified tissue correlates with the risk of developing adverse cardiac events in the future, such as myocardial infarction or sudden cardiac death. In addition it seems that CS is a better risk predictor for prospective cardiac events than currently available risk stratification score systems, such as Framingham or ATPIII risk score, in the way that CS reflects individual atherosclerotic disease burden. Especially the exclusion of coronary calcifications has been shown to exclude significant CAD in symptomatic patients [11-13]. However, the amount of calcium does not correlate well with stenosis severity [30]. Thus, aim of our study was to evaluate the use of an approach combining CS determination and SPECT-MPI. Especially we intended to identify the settings in which one can refrain from additional imaging or incremental information is won by the combination of both modalities.

In our patient population of 351 consecutive patients admitted for invasive coronary angiography, disease prevalence of significant CAD needing intervention was 38% reflecting a typical population undergoing ICA. As expected, specificity and positive predictive value of coronary calcium scoring were rather low. Thus, detection of hemodynamically relevant stenosis of epicardial vessels utilizing this modality is not practicable. However, a high sensitivity of 99.2%, and a high NPV of 98.5% suggest CS being a useful tool for ruling out CAD by exclusion of calcified plaque tissue. In these patients an additional SPECT-MPI did not further increase diagnostic accuracy as the negative predictive value of SPECT-MPI is limited to 92.1%. Following these results we would have correctly refrained from ICA and SPECT-MPI in 66 patients (18.8%) of our study population. Although there are some publications questioning the safety of a zero to low coronary calcium scores for the purpose of CAD exclusion in symptomatic patients, several studies in large patient cohorts consistently have shown a high NPV of >95% utilizing a threshold of zero calcium similar to our study. But also in these studies the safety of ruling out CAD is reduced by using higher thresholds like a CS of 10 or a CS above the 25th age and sex adjusted percentile instead of the exclusion of calcified tissue [11-13]. The same was true for our study: increasing CS thresholds would lead to an increase in specificity and PPV. Concomitantly sensitivity and NPV decreased to a value of 40.6% and 72.5%, respectively. Thus CS would no longer be suitable as a filter for invasive angiography and still not sufficient for the detection of relevant stenosis with a PPV of 84.4%. Still, diagnostic accuracy of CS at a threshold of 100 is comparable to the diagnostic performance of SPECT-MPI.

Due to low specificities of positive coronary calcium scores, a functional test like SPECT-MPI following initial CS in case of calcified tissue detection is reasonable. In our 284 patients with detectable coronary calcifications 51.1% (n=145) showed ischemia in SPECT-MPI followed by intervention in 80.0% (n=116) of those patients. Specificity of this combined approach increased to 81% with a disease prevalence of now 46.5%. Additionally a positive ischemic burden and its relation to a specific coronary vessel territory, provides helpful information for guiding interventional procedure.

In remaining 139 patients with positive calcium scores, SPECT-MPI excluded significant CAD in 123 patients (88.5%). Although CS did not help further with respect to CAD detection in these particular patients, it nonetheless provides individual prognostic information and estimates risk of developing prospective major coronary events. In that respect CS might outperform SPECT-MPI in symptomatic patients in the long-time follow-up [31]. An overall of 16 patients (11.5%) were misclassified by SPECT-MPI. Mean CS of these patients exceeded 300, with below 100 in only three cases, so that the majority of these patients presumably would have been put on secondary prevention therapy due to the CS results [32]. Furthermore it remains questionable whether these patients in particular would benefit from additional revascularization according to the nuclear medicine sub-study of the Clinical Outcomes Utilizing Revascularization and Aggressive Drug Evaluation (COURAGE) trial [33]. In this study Shaw et al. compared the effectiveness of adding coronary revascularization in order to minimize stress-induced ischemia with that of optimal medical therapy, utilizing SPECT-MPI to find the end point. They found that the outcome in patients with stable CAD was most strongly associated with the amount of residual ischemia after medical, or revascularization therapy, or both.

### Limitations

The utilization of different scanner technologies (MDCT and DSCT scanners) poses the risk of inconsistent calcium score values. According to a currently published head-to-head comparison of these two scanner types by Ghadri et al. revealed an excellent inter-scanner agreement for Agatston scores with a correlation coefficient of 0.976 despite relatively wide limits of agreement and a coefficient of variation 15.1% even in case of different vendors [34]. Calcium mass score as well as calcium volume score were also evaluated showing also excellent correlation, but wider limits of agreement in Bland-Altman Analysis. Interestingly the authors found that the use of different workstations had a greater influence on the comparability than the scanner technology. To account for those possible sources of error a comparable acquisition protocol for both scanner technologies routinely is used at our institution and the same workstation with the same software solution was utilized to calculate the Agatston score. Indication for revascularization was solely based on detection of significant stenosis (≥75%) in QCA and was independently made by the examiner without necessary verification of existing ischemia in order to guarantee for a blinded study design. This approach to revascularization procedure represents clinical practice at the time of study initiation. For comparison of SPECT-MPI a functional invasive measurement such as fractional flow reserve (FFR) would have been more appropriate, but FFR usually is not a widely available diagnostic modality. The use of the ≥75%-stenosis-degree criterion in order to define hemodynamically relevant CAD theoretically results in a higher number of false-positive findings with initially lower specificity and PPV. The likelihood of a ≥50%-stenosis causing ischemia, which serves as CAD defining endpoint in the majority of studies is relatively low and does not warrant revascularization procedure in the majority of cases unless prove of ischemic burden. Additionally selection bias must be considered while interpreting the data presented. First our evaluation was based on patients with clinical indication for ICA as referred by primary-care providers that do not necessarily sent unselected chest-pain patients for cardiac evaluation. Second our study was conducted at a single university site including only regional patients. Thus our study population may not reflect general symptomatic CAD patients.

## Conclusions

It was the aim of the study to evaluate the diagnostic accuracy of a combined approach using CS and SPECT-MPI. In patients with a CS=0 an additional SPECT-MPI did not lead to an increased diagnostic accuracy. The exclusion of CAD was attained with a high NPV by CS alone. In patients with a positive CS, SPECT-MPI increased specificity and PPV for the detection of CAD significantly.

In patients with a regular SPECT-MPI CS gave additional information on patients’ coronary atherosclerosis and cardiovascular risk.

## Abbreviations

CAC: coronary artery calcifications; CAD: coronary artery disease; CS: calcium scoring; CT: computed tomography; DA: diagnostic accuracy; ICA: invasive coronary angiography; MPI: myocardial perfusion imaging; NPV: negative predictive value; PET: positron emission tomography; PPV: positive predictive value; QCA: quantitative coronary analysis; SDS: summed difference score; SPECT: single photon emission computed tomography; SRS: summed rest scores; SSS: summed stress scores.

## Competing interests

The authors declare that they have no competing interests.

## Authors’ contributions

FvZ is responsible for coordination of the study, statistical evaluation and manuscript preparation. MB is responsible for data acquisition and statistical evaluation. CU and MH are responsible for SPECT-MPI data acquisition and evaluation. SH is responsible for data acquisition and data base maintenance. MG and JR are responsible for ICA studies. CB is responsible for CT scanning and CS evaluation. GS supervised ICA performance and interpretation. AB contributed in conception and design of the study and is responsible for manuscript review. All authors have read and approved the final manuscript.

## Pre-publication history

The pre-publication history for this paper can be accessed here:

http://www.biomedcentral.com/1471-2261/12/116/prepub
